# Dr. Bartholow’s Position

**Published:** 1886-09

**Authors:** 


### DR. BARTHOLOW'S POSITION.

An editorial in our July number, "Do we Need a Civil Service Reform in Medicine?" has elicited a letter from Dr. Roberts Bartholow, the distinguished Dean of Jefferson Medical College, which letter will be found in our Department of "Correspondence."

It will be seen that Dr. Bartholow strenuously denies that politics had anything to do with the removal of Drs. Atkinson and Shoemaker from the faculty. The Doctor gives us a refreshing little piece of news to the effect that he refused appointment under the original committee because he "had a confident anticipation that the arrangement would prove unsatisfactory because the organization did not have a sufficiently national character, in that it did not include many eminent men of the West and South." That is to say, he anticipated that the South and West would not submit to any such injustice, and would protest against it. He anticipated trouble, and stood from under. Had Dr. Bartholow, occupying the important position he does in the medical world, used his influence to have the organization *made* more representative, he would have shown himself to be a friend of the South and West, and of justice; and the result might have been different. His views are identical with those of Dr. Shoemaker, who promptly alligned himself in the direction of his sympathies and convictions, and spoke out, in defence of right. He thereby endeared himself to the South and West, and *they will not forget it.* It is said "a still tongue shows a wise head." Dr. Bartholow has given evidence of much wisdom, by this standard; he said nothing; and to-day the positions of the two gentlemen are very different; one is *in* and the other is *out* of Jefferson Medical College.

In reply to one portion of the Doctor's letter, we have to say that no "information" has been "given" us; nor have *we* made any charges. Our remarks were in the nature of a *surmise* as to the *cause* of the marked changes of the faculty. It looked like *more than a coincidence* that the discharged (and resigned) teachers should be on one side, and most of the faculty on the other side of the International Medical Congress question; and we thought we saw something like cause and effect. We had not seen the Baldwin-Bartholow correspondence at the time our editorial was written.

